# Supplementary material for: Biochemical Characterization of an Arabinoside Monophosphate Specific 5′-Nucleotidase-like Enzyme from Streptomyces antibioticus
Source: Biomolecules. 2024 Oct 27;14(11):1368. doi: 10.3390/biom14111368 (PMC11592144; doi:10.3390/biom14111368)
Supplement: Supplementary file 1 [file biomolecules-14-01368-s001.zip › biomolecules-3257712-supplementary.pdf]

Biochemical characterization of an arabinoside monophosphate specific 5'-nucleotidase-like enzyme from *Streptomyces antibioticus*

Yuxue Liu*, Xiaobei Liu, Xiaojing Zhang, Xiaoting Tang, Weiwei Su, Zhenyu Wang, and Hailei Wang*

Henan Engineering Research Center of Bioconversion Technology of Functional Microbes, College of Life Science, Henan Normal University, Xinxiang 453007, China;

* Corresponding author. E-mail address: liuyuxue@htu.edu.cn (Y. Liu); whl@htu.cn (H. Wang).

Table S1. The primers used in this study.

Oligonucleotide	Sequence (5′ to 3′)
R140A-F	CGTTGGCGCAGCGTTCGCGTTCGGCGGCCTG
R140A-R	GCGAACGCTGCGCCAACGCCAACCGCGCCCAT
F144A-F	GTTCGCGGCAGGCGGCCTGCTGGGTGAACC
F144A-R	GCCGCCTGCCGCGAACGCACGGCCAACGC
H174A-F	CTGGCGGCACTGTACGAAAAACTGGTTCACCTG
H174A-R	TCGTACAGTGCCGCCAGCACGGAGGAGGTTT
K178A-F	CTGTACGAAGCCCTGGTTCACCTGGAAAAAGACATGC
K178A-R	GGTGAACCAGGGCTTCGTACAGGTGCGCCAGCAC

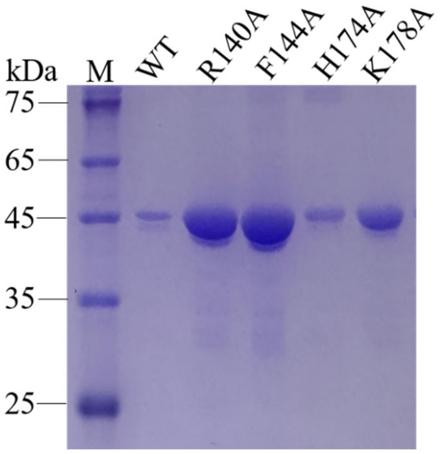

Figure S1. Analysis of purified of PenF and mutants by denaturing polyacrylamide gel electrophoresis.

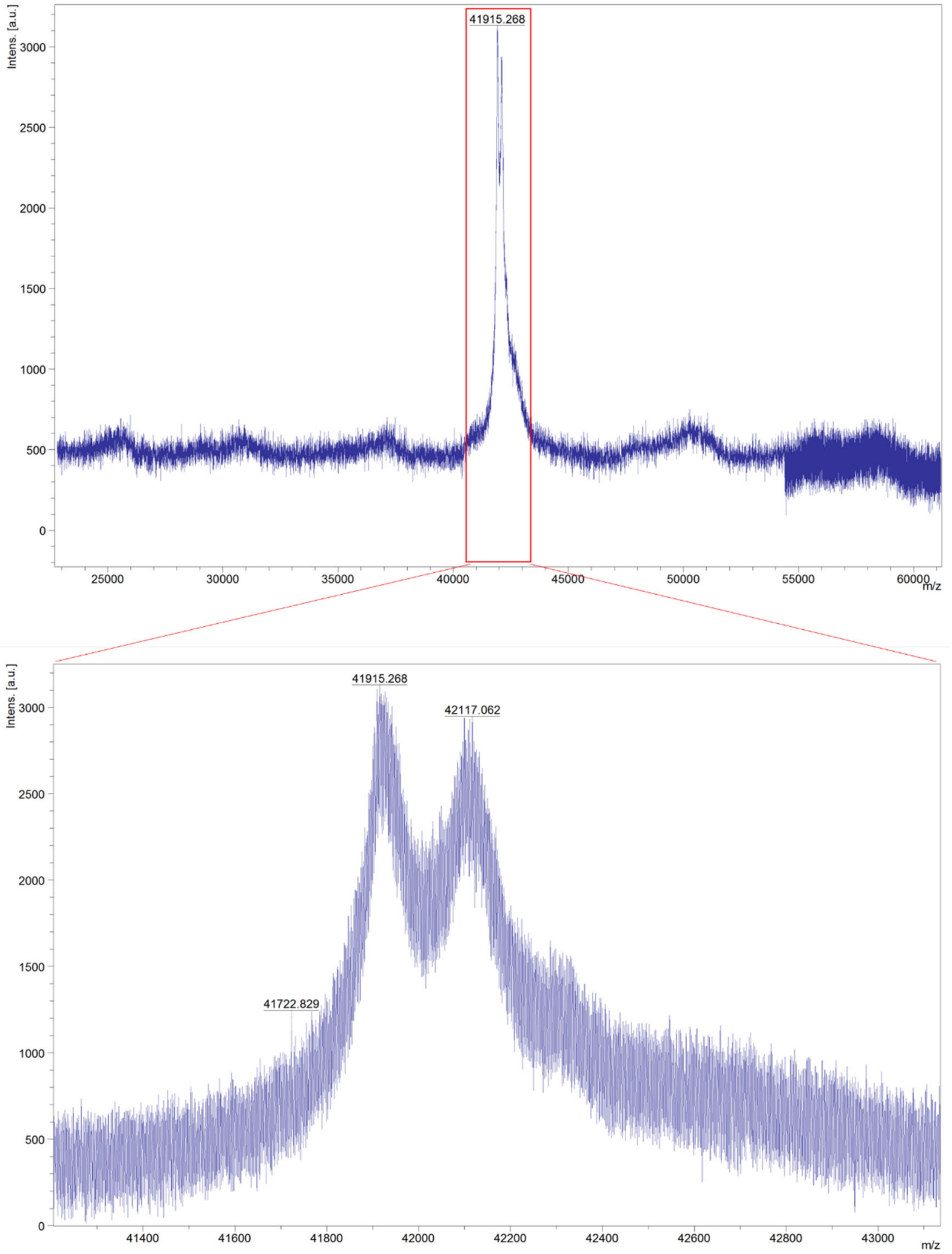

Figure S2. The matrix-assisted laser desorption ionization time-of-flightmass analysis of purified PenF.

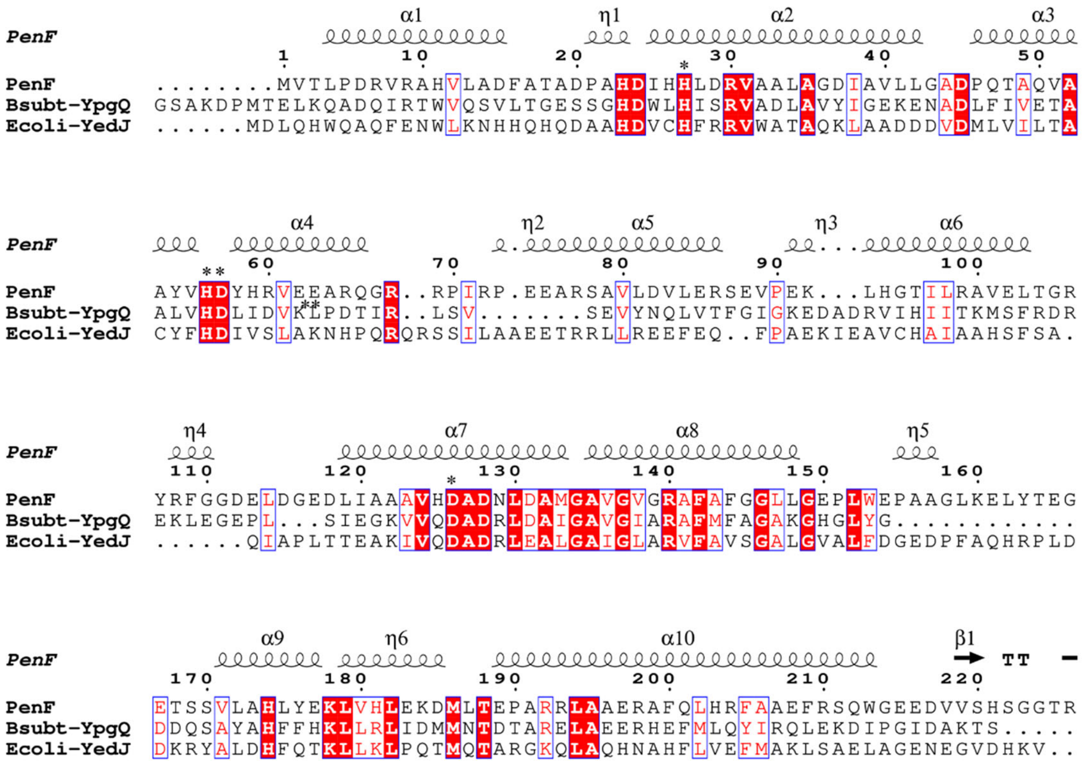

Figure S3. Multiple sequence alignment and secondary structure prediction of PenF with characterized HD-Domain phosphohydrolases. Bsubt-YpgQ, YpgQ from *Bacillus subtilis;* Ecoli-YedJ, YedJ from *E. coli.*

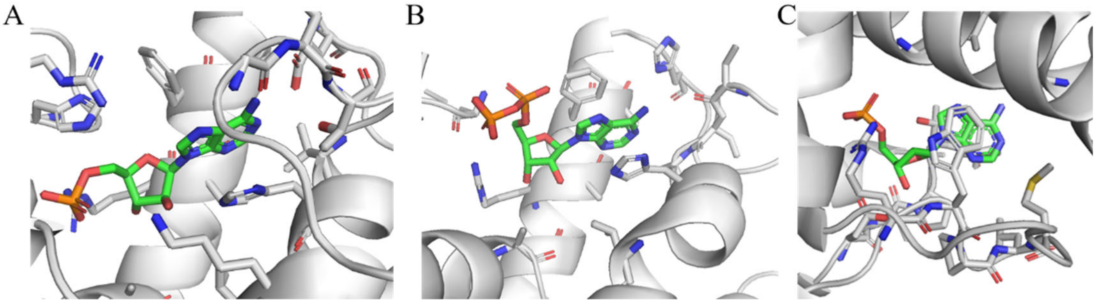

Figure S4. Close-up view of the ribose binding site of phosphohydrolases. (A) PenF in complex with Ara-AMP; (B) YpgQ in complex with ADP; (C) *E. coli* 5'-deoxynucleotidase YfbR in complex with dAMP.
